# Unveiling the Impact of the Omicron Variant: Insights from Genomic Surveillance in Mato Grosso do Sul, Midwest Brazil

**DOI:** 10.3390/v15071604

**Published:** 2023-07-22

**Authors:** Lívia de Mello Almeida Maziero, Marta Giovanetti, Vagner Fonseca, Marina Castilhos Souza Umaki Zardin, Gislene Garcia de Castro Lichs, Grazielli Rocha de Rezende Romera, Daniel Henrique Tsuha, Danila Fernanda Rodrigues Frias, Valdir Castanho Escandolhero, Luiz Henrique Demarchi, Larissa Domingues Castilho, Karine Ferreira Barbosa, Danielle Galindo Martins Tebet, Joilson Xavier, Hegger Fritsch, Mauricio Lima, Carla de Oliveira, Elaine Vieira Santos, Simone Kashima, Rodrigo Fabiano do Carmo Said, Alexander Rosewell, Julio Croda, Luiz Carlos Junior Alcantara, Crhistinne Cavalheiro Maymone Gonçalves

**Affiliations:** 1 Secretaria de Estado de Saúde de Mato Grosso do Sul, Campo Grande 9031-350, Brazil; mellolivia12@hotmail.com (L.d.M.A.M.); grazielli_r@hotmail.com (G.R.d.R.R.); daniel.tsuha@saude.ms.gov.br (D.H.T.); danila.frias@saude.ms.gov.br (D.F.R.F.); valdir.escandolhero@outlook.com (V.C.E.); larissacastilhodocs@gmail.com (L.D.C.); karinefbarbosa@gmail.com (K.F.B.); dani.rafatebet@gmail.com (D.G.M.T.); crhismay@gmail.com (C.C.M.G.); 2 Universidade Federal de EMato Grosso do Sul, Campo Grande 79070-900, Brazil; 3 Instituto Rene Rachou, Fundação Oswaldo Cruz, Preto 30190-009, Brazil; joilsonxavier@live.com (J.X.); hegger.fritsch@gmail.com (H.F.); maurili15@hotmail.com (M.L.); luiz.alcantara@fiocruz.br (L.C.J.A.); 4 Sciences and Technologies for Sustainable Development and One Health, University of Campus Bio-Medico of Rome, 00128 Rome, Italy; 5 Coordenação de Vigilância, Preparação e Resposta à Emergências e Desastres (PHE), Organização Pan-Americana da Saúde/Organização Mundial da Saúde (OPAS/OMS), Brasília 25045-002, Brazil; vagnerfonseca@gmail.com; 6 SES-MS—Laboratório Central de Saúde Pública de Mato Grosso do Sul, Campo Grande 79080-320, Brazil; ninaumaki@gmail.com (M.C.S.U.Z.); glichs@hotmail.com (G.G.d.C.L.); lhdemarchi@uol.com.br (L.H.D.); 7 lnstituto Oswaldo Cruz, Fundação Oswaldo Cruz, Rio de Janeiro 21040-900, Brazil; oliveirasc@yahoo.com.br; 8 Fundação Hemocentro de Ribeirão Preto, Rio de Janeiro 14015-160, Brazil; elainevs@alumni.usp.br (E.V.S.); skashima@hemocentro.fmrp.usp.br (S.K.); 9 Organização Pan-Americana da Saúde/Organização Mundial da Saúde (OPAS/OMS), Brasilia 25045-002, Brazil; saidrod@paho.org (R.F.d.C.S.); rosewelale@paho.org (A.R.); 10 Fundação Oswaldo Cruz, Universidade Federal de Mato Grosso do Sul—UFMS, Campo Grande 79081-746, Brazil; juliocroda@gmail.com

**Keywords:** SARS-CoV-2, Omicron variant, Mato Grosso do Sul, genomic monitoring

## Abstract

Genomic surveillance has emerged as a crucial tool in monitoring and understanding the dynamics of viral variants during the COVID-19 pandemic. In the Midwest region of Brazil, Mato Grosso do Sul has faced a significant burden from the SARS-CoV-2 epidemic, with a total of 613,000 confirmed cases as of June 2023. In collaboration with the Central Public Health Laboratory in the capital city of Campo Grande, we conducted a portable whole-genome sequencing and phylodynamic analysis to investigate the circulation of the Omicron variant in the region. The study aimed to uncover the genomic landscape and provide valuable insights into the prevalence and transmission patterns of this highly transmissible variant. Our findings revealed an increase in the number of cases within the region during 2022, followed by a gradual decline as a result of the successful impact of the vaccination program together with the capacity of this unpredictable and very transmissible variant to quickly affect the proportion of susceptible population. Genomic data indicated multiple introduction events, suggesting that human mobility played a differential role in the variant’s dispersion dynamics throughout the state. These findings emphasize the significance of implementing public health interventions to mitigate further spread and highlight the powerful role of genomic monitoring in promptly tracking and uncovering the circulation of viral strains. Together those results underscore the importance of proactive surveillance, rapid genomic sequencing, and data sharing to facilitate timely public health responses.

## 1. Introduction

The Severe Acute Respiratory Syndrome Coronavirus 2 (SARS-CoV-2) is a highly contagious coronavirus that emerged in late 2019, causing a global pandemic of acute respiratory illness known as ‘coronavirus disease 2019’ (COVID-19) and posing a significant threat to human health. The disease was first reported in Wuhan, Hubei province, China, with the World Health Organization declaring it a global public health emergency on 30 January 2020 [1,2]. Brazil confirmed its first case on 26 February 2020, followed by community transmission nationwide on 20 March 2020 [1,3]. Mato Grosso do Sul, situated in the Midwest region of Brazil, has experienced a substantial burden from the SARS-CoV-2 epidemic, with a total of 613,000 confirmed cases as of June 2023. As a crucial corridor for wildlife movement between different biomes, the state plays a pivotal role in maintaining biodiversity. Thus, implementing genomic surveillance can aid in identifying genetic interactions and gene flow between species, supporting conservation efforts, and preserving genetic integrity also considering that the Pantanal, one of the world’s largest tropical wetland areas, flourishes in Mato Grosso do Sul. In the context of the SARS-CoV-2 epidemic, the state has responded quickly by implementing various measures, such as social distancing, mask-use promotion, and localized lockdowns. Increased testing capacity and improved contact tracing efforts have also been essential [4]. Different variants, including variants of concern (VOCs), variants of interest (VOIs), and variants under monitoring (VUMs), have been identified in the state throughout the epidemic progression, [5]. However, the genomic diversity and the evolution dynamics of the SARS-CoV-2 Omicron wave in the state of Mato Grosso do Sul remain largely unknown. To address this gap, we conducted a collaborative study with the Central Public Health Laboratory in the capital city of Campo Grande. Our study involved portable whole-genome sequencing and phylodynamic analysis to investigate the spread and transmission patterns of the Omicron variant in the region. This research has provided insights into the expansion of this lineage in the state, revealing a complex transmission dynamic characterized by the co-circulation of different sublineages. In addition to shedding light on the Omicron variant, our study serves as a proof-of-concept for the value of portable sequencing technologies in local capacity building and public health efforts.

## 2. Materials and Methods

### 2.1. Sample Collection and Molecular Diagnostic Assays

Convenience clinical samples were collected between January and February 2022 from individuals suspected of SARS-CoV-2 infection and were included in our genomic surveillance framework for real-time monitoring of circulating SARS-CoV-2 variants in the region [5]. Nasopharyngeal swabs were utilized to extract viral RNA, which was subsequently subjected to analysis using the Charité SARS-CoV2 (E/RP) assay provided by Bio-Manguinhos. This assay specifically targeted the E gene and was made available by the Brazilian Ministry of Health (BrMoH) and the Pan-American Health Organization. The samples were collected for both diagnostic purposes and whole genome sequencing analysis.

### 2.2. cDNA Synthesis and Whole-Genome Sequencing

Samples were chosen for sequencing based on a Ct value (≤30) and the availability of epidemiological metadata, including the date of sample collection, sex, age, and municipality of residence. The SARS-CoV-2 genomic libraries were prepared using nanopore sequencing. Complementary DNA (cDNA) synthesis was performed using the SuperScript IV Reverse Transcriptase kit (Invitrogen, Waltham, MA, USA) following the manufacturer’s instructions. The generated cDNA underwent multiplex PCR sequencing using the Q5 High-Fidelity Hot-Start DNA Polymerase (New England Biolabs, Ipswich, MA, USA) and a set of specific primers designed by the ARTIC Network for sequencing the complete SARS-CoV-2 genome (Artic Network version 3) (Quick, J, 2020). PCR conditions have been previously reported [3]. All experiments were conducted within a biosafety level 2 cabinet. Amplicons were purified using 1× AMPure XP beads (Beckman Coulter, Pasadena, CA, USA) and quantified with a Qubit 3.0 fluorimeter (ThermoFisher, Waltham, MA, USA) using the Qubit dsDNA HS assay kit (ThermoFisher). DNA library preparation was carried out using the ligation sequencing kit LSK109 (Oxford Nanopore Technologies, Oxford, UK) and the native barcoding kit (NBD104 and NBD114, Oxford Nanopore Technologies). The prepared sequencing libraries were loaded onto an R9.4 flow cell (Oxford Nanopore Technologies). In each sequencing run, negative controls were included to prevent and detect possible contamination, with a mean coverage of less than 2%.

### 2.3. Generation of Consensus Sequences

The raw files from Oxford Nanopore sequencing were basecalled using Guppy v3.4.5, and barcode demultiplexing was conducted using qcat. Consensus sequences were then generated through de novo assembly using Genome Detective [6].

### 2.4. Phylogenetic Analysis

Lineage assignment was conducted using the Phylogenetic Assignment of Named Global Outbreak Lineages tool (PANGOLIN) [7]. The newly generated sequences from this study were compared to a diverse pool of 2986 genome sequences collected worldwide up until 2 June 2023. Due to the extensive amount of available data and the uneven distribution of strains from different regions, countries, and continents, we utilized the Subsampler tool, available at https://github.com/andersonbrito/subsampler (accessed on 10 July 2023), which enabled us to perform random subsampling of sequences per country based on case counts over the study period. By doing so, we ensured that our samples were geographically, temporally, and epidemiologically representative. Furthermore, the subsampling within this scheme was conducted using a baseline function, which allowed us to determine the proportion of cases we aimed to sample. This process was carefully designed to maintain the overall characteristics and diversity of the data while minimizing potential biases. All sequences were aligned using the ViralMSA tool [8] and the maximum likelihood approach was employed for phylogenetic analysis using IQ-TREE 2 [9]. To obtain a dated tree, TreeTime [10] was employed, employing a constant mean rate of 8.0 × 10^−4^ nucleotide substitutions per site per year, after removing outlier sequences.

### 2.5. Epidamiological Data Assesment

Data from weekly notified cases of infection, deaths, and hospitalization by SARS-CoV-2 in the state of Mato Grosso do Sul were supplied by the Brazilian Ministry of Health, as made available by the COVIDA network at https://github.com/wcota/COVID19br (accessed on 10 July 2023).

## 3. Results

**Figure 1:**
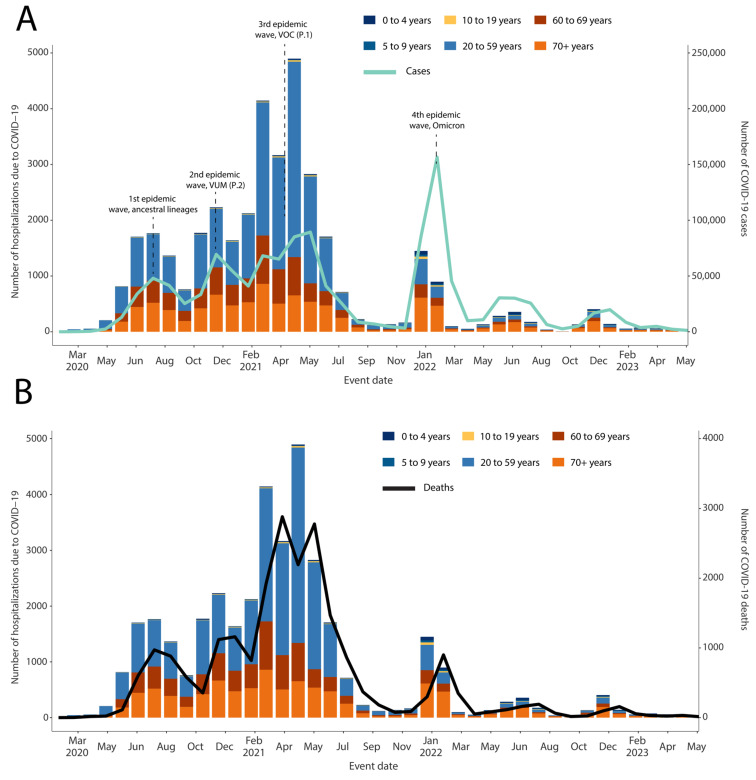
Dynamics of the SARS-CoV-2 epidemic in the state of Mato Grosso do Sul. (**A**) Number of daily COVID-19 cases and the number of hospitalization rates in time; (**B**) number of daily COVID-19 deaths and the number of hospitalization rates in time.

**Figure 2:**
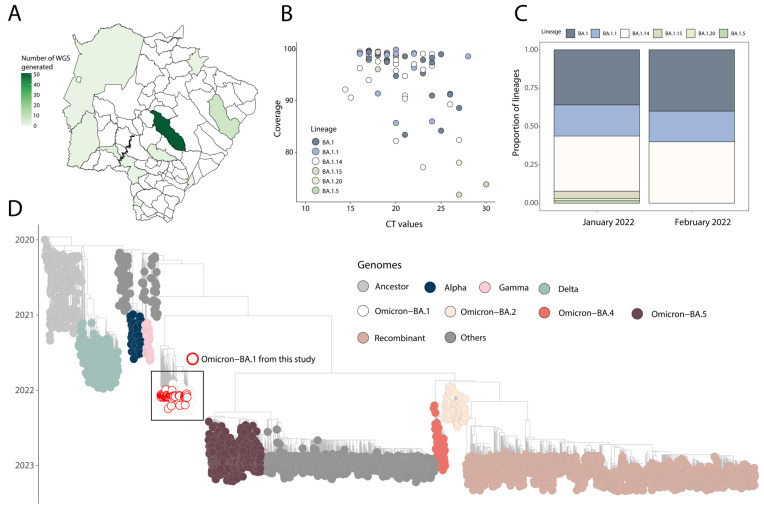
Genomics and epidemiological reconstruction of SARS-CoV-2 Omicron sublineages circulating in the state of Mato Grosso do Sul. (**A**) Spatial distribution of SARS-CoV-2 genomes obtained in this study; (**B**) SARS-CoV-2 sequencing statistics: percentage of SARS-CoV-2 genomes sequenced plotted against RT-qPCR Ct value for each sample (n = 69). Each circle represents a sequence recovered from an infected individual in Mato Grosso do Sul. Colored circles correspond to lineage assignment; (**C**) progressive distribution of SARS-CoV-2 BA.1 sublineages in the state of Mato Grosso do Sul over time; (**D**) time-resolved maximum-likelihood tree of SARS-CoV-2, including a representative worldwide subsample of genomes (n = 2986) collected up to 2 June 2023. The genomes are color-coded according to lineages (VOC and ancestral lineages) as indicated in the legend on the top right. The genome generated in this study is highlighted in the tree with a white fill and a red circle.

The SARS-CoV-2 epidemic in the state of Mato Grosso do Sul consisted of four distinct main waves (Figure 1), resulting in over 613,000 thousand cases and 11 thousand deaths as of June 2023. The initial wave spanned from May 2020 to September 2020 and exhibited the circulation of various ancestral lineages (Figure 1 and Figure 2) [3].

The second wave, which occurred from October 2020 to December 2020, was driven by the emergence and rapid spread of the P.2 (Zeta) variant under monitoring (VUM). In contrast, the third wave (December 2020 to May 2021) was primarily caused by the introduction of the Gamma (P.1) variant of concern (VOC), resulting in a significant increase in the number of reported cases and deaths in the country [11]. However, as the Delta VOC was introduced in late April 2021, the number of cases and deaths started to decline (Figure 1) [12]. Following these waves, the fourth major wave occurred in late November 2021 with the emergence of the Omicron variant as the newest VOC. Our data suggest that the Omicron wave experienced a rapid peak followed by a swift decline, possibly indicating the successful impact of the vaccination program [13]. Additionally, the highly transmissible nature of the Omicron variant played a significant role in swiftly affecting a large portion of the susceptible population.

Furthermore, our analysis of the total number of cases, deaths, and COVID-19-related hospitalizations revealed that the age group between 20 and 59 years bore the highest disease burden from February to May 2021. This spike in cases coincided with the circulation of the Gamma variant, which overwhelmed healthcare facilities at both the national and regional levels, primarily impacting the susceptible population.

In order to retrospectively reconstruct the transmission dynamic of the Omicron variant in the state of Mato Grosso do Sul, a total of 69 near-full genome sequences were obtained from SARS-CoV-2 RT-qPCR positive samples as part of this study. The sequencing spanned from January to February 2022, and samples were collected from 10 distinct cities across the state (Figure 2A).

These samples comprised 41 females and 28 males (Table 1), with a median age of 41.0 years (range: 11 to 91 years).

All tested samples contained sufficient viral genetic material (≥2 ng/µL) for library preparation. The average PCR cycle threshold (Ct) value for positive samples was 20.75 (range: 10 to 30). Sequences had a median genome coverage of 94% (range: 72 to 99.99), with samples that had lower Ct values generally exhibiting higher average genome coverage (Figure 2B). Detailed epidemiological information and sequencing statistics of the generated sequences can be found in Table 1. Based on the proposed dynamic nomenclature for SARS-CoV-2 lineages, the sequences were assigned to six different Omicron BA.1 PANGO-lineages (Figure 2C and Table 1). Novel genome sequences have been submitted to GISAID following the WHO guidelines (Table 1) (version 4.2). Phylogenetic inference, combining our novel isolates with a representative dataset available on GISAID (https://www.gisaid.org/) up to 2 June 2023, revealed that the newly obtained genomes belong to different SARS-CoV-2 Omicron BA.1 sublineages (Figure 2D), which were interspersed with those introduced from several countries (Figure 2D). This pattern indicates multiple introduction events highlighting how critical integrating genome sequence with human mobility data will be to promptly reconstruct and track the transmission dynamics of those emerging strains. (Figure 1 and Figure 2).

## 4. Discussion

Genomic surveillance is of pivotal importance in monitoring SARS-CoV-2 variants and informing public health responses. Variants of concern (VOCs) have had a significant impact on the severity of COVID-19, therapeutic approaches, and vaccination efforts [3,14,15,16,17]. These variants are characterized by increased transmissibility and the potential to affect disease severity. Specific mutations, such as N501Y and E484K, have been identified as contributing to enhanced transmissibility and immune evasion. Therefore, continuous monitoring and regular updates in therapeutic strategies are essential to effectively address these evolving variants.

To assess the impact of the Omicron variant in Mato Grosso do Sul, a state located in the Midwest region of Brazil, we conducted on-site training in genomic surveillance in collaboration with the state’s Public Health Laboratory. Through this collaboration, we were able to generate 69 newly SARS-CoV-2 complete genome sequences. Our analysis revealed four waves of the SARS-CoV-2 epidemic in the state, with each wave characterized by the circulation and replacement of different viral strains [11,17]. Consistent with previous findings, the Gamma variant was associated with a significant increase in reported cases and deaths in the state, particularly among individuals aged 20–59 (Figure 1).

Our data demonstrated that the emergence of the Omicron variant coincided with the fourth major wave, exhibiting a distinct pattern of a rapid peak followed by a swift decline (Figure 1). This trend can be attributed to several factors. The vaccination program played a crucial role in reducing illness severity and transmission, as a significant portion of the population had received vaccination doses by the time the Omicron variant emerged. The highly transmissible nature of the Omicron variant contributed to exponential growth in cases, but the number of susceptible individuals decreased due to vaccination or prior infection [18].

Genomic monitoring in our study additionally identified distinct Omicron sublineages (Table 1 and Figure 2), indicating complex transmission dynamics influenced by the co-circulation of multiple viral strains. Human mobility played a significant role in the dissemination of these sublineages, facilitated by Mato Grosso do Sul’s geographical location as a border region and active trade and travel routes [19]. Understanding the impact of human mobility on variant introduction and dissemination is crucial for effective public health interventions.

In conclusion, this study emphasizes the significance of genomic monitoring in comprehending the transmission dynamics of emerging variants. Further advancement in the field of genomic surveillance necessitates investigation in several areas and should focus on long-term genomic monitoring to track the evolutionary trajectory and potential changes in viral genetic characteristics. Comparative genomic analyses across different regions and countries will also be crucial to identify geographic-specific patterns and inform targeted public health interventions. Exploring the role of host genetic factors and their interplay with viral evolution will further enhance our understanding of susceptibility, disease severity, and vaccine response. Lastly, expanding genomic surveillance to include animal hosts and environmental samples in a One Health approach can facilitate early detection and response to zoonotic transmission.

## Figures and Tables

**Table 1:** Epidemiological data the 69 SARS-CoV-2 samples sequenced as part of this study.

ID	CT	State	Collection Date	Sex	Age	Reads	Coverage	Depth of Coverage	Lineage	Accession ID
LIBSARSMS2_barcode12|2022-01-24	23	MS	24 January 2022	M	48	60,759	99.1	1104	BA.1.14	EPI_ISL_17885390
LIBSARSMS2_barcode16|2022-02-11	21	MS	28 January 2022	F	57	7159	91	141.7	BA.1.14	EPI_ISL_17885391
LIBSARSMS2_barcode17|2022-01-27	25	MS	27 January 2022	F	43	91,596	99	1666.2	BA.1	EPI_ISL_17885392
LIBSARSMS2_barcode19|2022-01-24	21	MS	25 January 2022	F	11	103,201	98.6	1885.8	BA.1	EPI_ISL_17885393
LIBSARSMS2_barcode20|2022-01-25	27	MS	25 January 2022	M	13	3271	71.8	79.4	BA.1.15	EPI_ISL_17885394
LIBSARSMS2_barcode21|2022-01-25	27	MS	25 January 2022	M	34	5427	82.4	119.8	BA.1.14	EPI_ISL_17885395
LIBSARSMS2_barcode22|2022-01-25	18	MS	25 January 2022	F	18	138,324	98.8	2606.4	BA.1	EPI_ISL_17885396
LIBSARSMS2_barcode23|2022-01-25	24	MS	25 January 2022	F	25	15,665	91	349.3	BA.1	EPI_ISL_17885397
LIBSARSMS2_barcode25|2022-01-27	18	MS	26 January 2022	F	20	82,194	96.1	1525.3	BA.1.15	EPI_ISL_17885398
LIBSARSMS2_barcode26|2022-01-26	23	MS	26 January 2022	F	74	54,957	95.3	1037	BA.1	EPI_ISL_17885399
LIBSARSMS2_barcode27|2022-01-28	22	MS	27 January 2022	M	52	69,658	99.2	1261.8	BA.1.14	EPI_ISL_17885400
LIBSARSMS2_barcode28|2021-12-29	24	MS	27 January 2022	M	54	63,882	99	1162.4	BA.1.14	EPI_ISL_17885401
LIBSARSMS2_barcode29|2022-02-03	24	MS	26 January 2022	F	61	36,138	96.8	672.6	BA.1.14	EPI_ISL_17885402
LIBSARSMS2_barcode30|2022-02-04	25	MS	26 January 2022	F	57	6383	84.2	135.5	BA.1	EPI_ISL_17885403
LIBSARSMS2_barcode32|2022-02-04	27	MS	26 January 2022	M	37	8017	88.6	157.5	BA.1	EPI_ISL_17885404
LIBSARSMS2_barcode34|2022-01-20	21	MS	26 January 2022	M	24	15,481	83.4	331.3	BA.1	EPI_ISL_17885405
LIBSARSMS2_barcode35|2022-02-03	20	MS	26 January 2022	M	34	47,285	97.9	870.2	BA.1	EPI_ISL_17885406
LIBSARSMS2_barcode36|2022-02-03	19	MS	26 January 2022	M	35	58,617	97.9	1082.5	BA.1	EPI_ISL_17885407
LIBSARSMS2_barcode37|2022-02-03	17	MS	26 January 2022	F	37	199,034	99.7	3576.7	BA.1	EPI_ISL_17885408
LIBSARSMS2_barcode38|2022-02-03	20	MS	26 January 2022	M	53	85,187	98.8	1561.2	BA.1	EPI_ISL_17885409
LIBSARSMS2_barcode39|2022-01-31	27	MS	27 January 2022	F	20	2722	78	60.3	BA.1.20	EPI_ISL_17885410
LIBSARSMS2_barcode41|2022-02-04	21	MS	27 January 2022	F	54	78,674	94.8	1483.9	BA.1.14	EPI_ISL_17885411
LIBSARSMS2_barcode42|2022-02-04	17	MS	27 January 2022	F	37	77,568	97.9	1429.8	BA.1.1	EPI_ISL_17885412
LIBSARSMS2_barcode43|2022-02-04	26	MS	27 January 2022	F	54	12,672	91.3	248.5	BA.1	EPI_ISL_17885413
LIBSARSMS2_barcode44|2022-02-04	18	MS	27 January 2022	M	21	164,942	99.5	2957.3	BA.1.1	EPI_ISL_17885414
LIBSARSMS2_barcode45|2022-02-04	17	MS	27 January 2022	M	19	141,215	99.5	2541.5	BA.1.14	EPI_ISL_17885415
LIBSARSMS2_barcode46|2022-02-04	20	MS	27 January 2022	M	64	112,016	99.9	2027.6	BA.1.1	EPI_ISL_17885416
LIBSARSMS2_barcode47|2022-03-17	20	MS	28 January 2022	M	57	23,799	95.8	446.7	BA.1.14	EPI_ISL_17885417
LIBSARSMS2_barcode48|2022-01-29	19	MS	28 January 2022	F	19	166,009	99.3	2998.2	BA.1.1	EPI_ISL_17885418
LIBSARSMS2_barcode49|2022-01-27	18	MS	27 January 2022	F	65	20,051	91.4	398.1	BA.1.1	EPI_ISL_17885419
LIBSARSMS2_barcode50|2022-01-27	19	MS	27 January 2022	F	31	121,892	99	2209.4	BA.1.14	EPI_ISL_17885420
LIBSARSMS2_barcode51|2022-01-27	17	MS	27 January 2022	F	54	188,893	98.7	3464.9	BA.1.14	EPI_ISL_17885421
LIBSARSMS2_barcode52|2022-01-27	18	MS	27 January 2022	M	24	81,577	99.5	1477.2	BA.1	EPI_ISL_17885422
LIBSARSMS2_barcode53|2022-01-27	18	MS	27 January 2022	F	36	160,723	99.2	2908.4	BA.1	EPI_ISL_17885423
LIBSARSMS2_barcode55|2022-01-27	19	MS	27 January 2022	M	43	28,809	97.6	533.8	BA.1	EPI_ISL_17885424
LIBSARSMS2_barcode56|2022-01-27	16	MS	27 January 2022	M	42	54,509	96.3	1005.8	BA.1.14	EPI_ISL_17885425
LIBSARSMS2_barcode57|2022-01-27	19	MS	27 January 2022	M	35	64,896	97.5	1187.5	BA.1	EPI_ISL_17885426
LIBSARSMS2_barcode58|2022-01-21	20	MS	28 January 2022	F	31	68,573	94.8	1309.9	BA.1	EPI_ISL_17885427
LIBSARSMS2_barcode59|2022-01-28	20	MS	28 January 2022	M	37	77,921	97.1	1436.8	BA.1.14	EPI_ISL_17885428
LIBSARSMS2_barcode60|2022-01-25	30	MS	25 January 2022	F	32	1494	73.8	33.8	BA.1.5	EPI_ISL_17885429
LIBSARSMS2_barcode61|2022-01-31	18	MS	31 January 2022	F	65	180,831	99.4	3270.9	BA.1.15	EPI_ISL_17885430
LIBSARSMS2_barcode62|2022-01-28	24	MS	28 January 2022	F	16	36,276	97.4	674.6	BA.1	EPI_ISL_17885431
LIBSARSMS2_barcode63|2022-01-27	19	MS	27 January 2022	F	62	74,361	98.9	1364.4	BA.1.1	EPI_ISL_17885432
LIBSARSMS2_barcode65|2022-01-27	28	MS	28 January 2022	F	60	198,829	98.6	3652.5	BA.1.1	EPI_ISL_17885433
LIBSARSMS2_barcode66|2022-02-02	23	MS	2 February 2022	F	14	13,165	77.1	312.2	BA.1.14	EPI_ISL_17885434
LIBSARSMS2_barcode67|2022-02-02	23	MS	2 February 2022	M	39	33,181	97.9	611.9	BA.1	EPI_ISL_17885435
LIBSARSMS2_barcode68|2022-01-18	20	MS	31 January 2022	F	30	11,165	85.7	234.2	BA.1.1	EPI_ISL_17885436
LIBSARSMS2_barcode69|2022-03-15	16	MS	30 January 2022	M	27	266,105	99.5	4833.1	BA.1.14	EPI_ISL_17885437
LIBSARSMS2_barcode70|2022-03-13	20	MS	30 January 2022	F	26	112,571	98.6	2052.6	BA.1.1	EPI_ISL_17885438
LIBSARSMS2_barcode71|2022-02-07	20	MS	31 January 2022	F	91	209,974	99.1	3941.7	BA.1.1	EPI_ISL_17885439
LIBSARSMS2_barcode72|2022-03-31	15	MS	31 January 2022	F	91	35,836	90.6	712.6	BA.1.14	EPI_ISL_17885440
LIBSARSMS2_barcode73|2022-01-31	24	MS	31 January 2022	M	79	27,752	86	578.3	BA.1.1	EPI_ISL_17885441
LIBSARSMS2_barcode74|2022-01-31	17	MS	30 January 2022	M	23	76,270	94	1467	BA.1.14	EPI_ISL_17885442
LIBSARSMS2_barcode76|2022-01-24	20	MS	1 February 2022	F	39	20,573	82.2	452.5	BA.1.14	EPI_ISL_17885443
LIBSARSMS2_barcode78|2022-02-04	16	MS	1 February 2022	F	29	177,714	99.2	3249.3	BA.1	EPI_ISL_17885444
LIBSARSMS2_barcode79|2022-02-04	22	MS	1 February 2022	F	23	14,615	96.4	272.6	BA.1.1	EPI_ISL_17885445
LIBSARSMS2_barcode80|2022-01-29	21	MS	29 January 2022	F	52	11,173	90.4	221	BA.1.14	EPI_ISL_17885446
LIBSARSMS2_barcode81|2022-01-29	24	MS	29 January 2022	M	62	41,745	98.3	763.3	BA.1	EPI_ISL_17885447
LIBSARSMS2_barcode82|2022-01-29	26	MS	29 January 2022	F	63	13,046	89.3	264.1	BA.1.14	EPI_ISL_17885448
LIBSARSMS2_barcode83|2022-01-29	22	MS	29 January 2022	F	49	35,076	99.1	631.7	BA.1.1	EPI_ISL_17885449
LIBSARSMS2_barcode84|2022-01-29	17	MS	29 January 2022	M	34	218,985	98.2	3999.7	BA.1	EPI_ISL_17885450
LIBSARSMS2_barcode86|2022-01-29	23	MS	29 January 2022	F	51	16,567	96	312.1	BA.1.14	EPI_ISL_17885451
LIBSARSMS2_barcode87|2022-01-29	10	MS	29 January 2022	M	39	11,270	88.6	224.4	BA.1.14	EPI_ISL_17885452
LIBSARSMS2_barcode88|2022-01-29	18	MS	29 January 2022	M	19	60,836	97.6	1112	BA.1.14	EPI_ISL_17885453
LIBSARSMS2_barcode90|2022-01-29	17	MS	29 January 2022	F	22	225,902	99.4	4159.7	BA.1	EPI_ISL_17885454
LIBSARSMS2_barcode91|2022-01-29	26	MS	29 January 2022	F	27	24,272	91.2	471.3	BA.1	EPI_ISL_17885455
LIBSARSMS2_barcode93|2022-01-29	20	MS	29 January 2022	M	55	66,819	97.9	1331.5	BA.1.14	EPI_ISL_17885456
LIBSARSMS2_barcode94|2022-01-18	16	MS	29 January 2022	F	30	203,956	99.1	4029.9	BA.1.1	EPI_ISL_17885457
LIBSARSMS2_barcode95|2022-01-01	22	MS	29 January 2022	F	40	20,911	97.1	389.3	BA.1.14	EPI_ISL_17885458

## Data Availability

Newly generated SARS-CoV2-sequences have been deposited in GISAID under accession numbers EPI_ISL_17885390 and EPI_ISL_17885458. All inputs and codes used in this study were made available in main text and on the project GitHub repository: https://github.com/genomicsurveillance/omicron_mato_grosso_do_sul/tree/main (accessed on 10 July 2023).
